# Permutation test for periodicity in short time series data

**DOI:** 10.1186/1471-2105-7-S2-S10

**Published:** 2006-09-26

**Authors:** Andrey A Ptitsyn, Sanjin Zvonic, Jeffrey M Gimble

**Affiliations:** 1 Experimental Obesity Laboratory, Louisiana State University Pennington Biomedical Research Center, 6400 Perkins Rd., Baton Rouge, LA 70808, USA; 2 Stem Cell Laboratory, Louisiana State University Pennington Biomedical Research Center, 6400 Perkins Rd., Baton Rouge, LA 70808, USA

## Abstract

**Background:**

Periodic processes, such as the circadian rhythm, are important factors modulating and coordinating transcription of genes governing key metabolic pathways. Theoretically, even small fluctuations in the orchestration of circadian gene expression patterns among different tissues may result in functional asynchrony at the organism level and may contribute to a wide range of pathologic disorders. Identification of circadian expression pattern in time series data is important, but equally challenging. Microarray technology allows estimation of relative expression of thousands of genes at each time point. However, this estimation often lacks precision and microarray experiments are prohibitively expensive, limiting the number of data points in a time series expression profile. The data produced in these experiments carries a high degree of stochastic variation, obscuring the periodic pattern and a limited number of replicates, typically covering not more than two complete periods of oscillation.

**Results:**

To address this issue, we have developed a simple, but effective, computational technique for the identification of a periodic pattern in relatively short time series, typical for microarray studies of circadian expression. This test is based on a random permutation of time points in order to estimate non-randomness of a periodogram. The *Permutated time*, or Pt-test, is able to detect oscillations within a given period in expression profiles dominated by a high degree of stochastic fluctuations or oscillations of different irrelevant frequencies. We have conducted a comprehensive study of circadian expression on a large data set produced at PBRC, representing three different peripheral murine tissues. We have also re-analyzed a number of similar time series data sets produced and published independently by other research groups over the past few years.

**Conclusion:**

The Permutated time test (Pt-test) is demonstrated to be effective for detection of periodicity in short time series typical for high-density microarray experiments. The software is a set of C++ programs available from the authors on the open source basis.

## Background

Circadian, or approximately daily, rhythm is one of the most well studied periodic processes in living organisms. Multiple studies in different tissues report that expression of approximately 5–15% of all genes show circadian oscillations [1,2]. Circadian oscillation is driven by a molecular mechanism involving a few genes co-regulated by self-sustaining feedback loops, which generate the basic rhythm driving their expression. In mammals the master circadian clock is located in the hypothalamus, and its oscillation is constantly adjusted to the daily light cycle through photic stimuli from the retina. Analysis of gene expression in peripheral tissues indicates that, while peripheral circadian mechanisms play an important role in different organs, relatively few genes share the same circadian expression profile in more then one tissue [3].

Identification of periodically expressed genes presents a significant challenge. Most of the statistical methods for detection of periodicity in timeline data have been developed for much longer series than those available for computational analysis of gene expression. Production of long time-series microarray experiments is prohibitively expensive. Even a single time series of 12 time points with a minimal number of replicates costs thousands of dollars in microarray chips, equipment and labor. Among publicly available data sets, few extend beyond 24 time points or cover more than 2 complete cycles. High variability and low expression level for many genes are additional challenges, further obscuring the periodicity of the base expression. The current manuscript describes a permutated time test designed to address these challenges.

## Results

### The permutation of time algorithm

Our alternative test for significance of a particular periodicity (in our case circadian) among large numbers of gene expression profiles is based on random permutation of time points, thus abbreviated as the *Pt*-test. To present this approach, consider a time series *Y *= *x*_0_, *x*_1_, *x*_2_, ...*x*_*N*-1_, in which technical variation approaches or even exceeds the amplitude of periodic expression. In a very short time series stochastic noise often obscures periodicity. However, the periodic change of the base expression level can still be identified in spite of the high noise level. If the periodogram of the original time series *IY(ω) *contains a significant peak corresponding to a particular frequency (for example, circadian) this peak results from a particular order of observation is the *Y*. A random permutation would preserve the same noise level, but not the periodicity. Let *YR *be a random permutation of the time series *Y*. Its corresponding periodogram is *IR(ω)*. After DFT a periodogram *IR(ω) *would represent only the peaks occurring by chance. However it will miss the true periodic frequencies unless permutations happen to preserve the period, for example if the rank of each point *x *in permutated series *YR *is equal *x*_*Y *_± n* *p *where *n *is a natural number and *p *is a period corresponding to a significant peak in *IY(ω)*. To avoid random re-institution of periodicity we generate *YR *by multiple shuffling of randomly selected time points *x*_*n *_⇔ *x*_*m*_, where |*n *- *m*| ≠ *p*, i.e. each shuffle is swaps time points from different phase. Comparing permutations with deliberately wiped out periodicity to the original time series we can estimate whether a particular order of observations (i.e. time series) is important. For each gene expression profile we generate two series of *min(n!,100) *random permutations. Each permutated series *YR *is transformed to the frequency domain and a single peak of the periodogram *IR(ω) *is stored. The p-value for the null-hypothesis of random nature of a particular peak of periodogram can be estimated by comparing the stored *IR(ω) *values to the observed *I(ω)*:



High *p*-value exceeding the threshold, for example 0.05, means that at least 5 out of 100 random permutations of time series produce a periodogram with the same or higher peak, corresponding to a given periodicity. Low *p*-values indicate a significant difference between periodogram *IR(ω) *preserving circadian periodicity and randomly permutated periodogram *IY(ω) *with the same level of technical variation. This difference leads to rejection of the null-hypothesis of purely random nature of variation in the original time series *Y*.

### Testing and benchmarking

Permutation is a commonly used procedure applied for estimation of *p*-value. Algorithms to recognize a circadian oscillatory pattern in Storch et al. and Panda et al. as well as many other papers employ some form of permutation. The principal difference of our test is that while we, like others, generate permutations in the time domain, we then analyze the periodograms of the permutated time series in the frequency domain. Since we consider only one frequency (in this study circadian, i.e. making 2 complete cycles in 12 time points series), stochastic variations between time points have a reduced impact compared to the time-domain methods. Fisher's g-test is based on the estimation of signal to noise ratio from the periodogram of a time series (i.e. in frequency domain). It is less effective on very short time series, such as those commonly found in functional genomics studies. In such time series, typically derived from a dozen or two microarray experiments, stochastic deviations can easily exceed the underlying baseline variation, which results in high peaks for irrelevant non-circadian frequencies and an overall poor resolution capability of the method. We have developed the Pt-test for short time series in order to combine the benefits of the frequency domain analysis with robust error rate estimation based on a permutation procedure.

**Figure 1:**
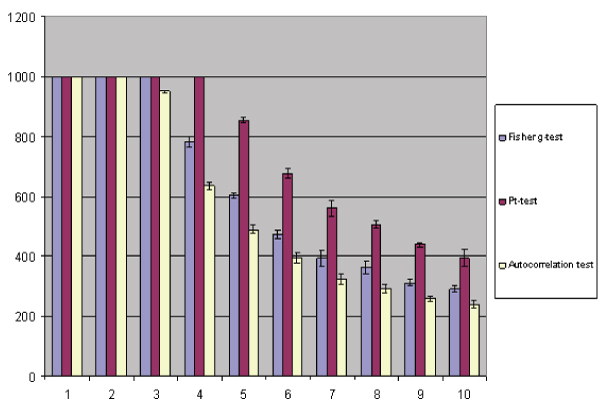
**Result of analysis of an artificial data set**. Result of analysis of an artificial data set. With increase of stochastic noise all three algorithms recognize fewer oscillating profiles. However in each category permutation test consistently report oscillation in more profiles than the other two algorithms. Bars delimit standard deviation of mean number of identified oscillating profiles based on 5 repeated simulations.

The results of our analysis of the simulated data set with 3 different algorithms are presented on Figure 1. When applied to the simulated data with controlled properties, none of the tested algorithms have reported a significant number of false positive oscillating profiles among profiles with 0 amplitude baseline. As expected, all algorithms report progressively fewer oscillating profiles as there is an increasing range of the stochastic noise component *ε *and a decreasing signal to noise ratio. In the first 2 categories (*ε *= 0 and *ε *∈ [0, 50] respectively) all three algorithms identify 100% profiles as oscillating. In every other category our permutation test tends to find more oscillating expression profiles compared to Fisher's g-test and autocorrelation.

To test the effectiveness of the new algorithm on real data we have re-analyzed three independent data sets. All of them represent murine liver time series over the equivalent of 2 complete daily cycles (48 hours). All three utilize Affymetrix GeneChips. However the data was acquired in different years by 3 different research institutes in independently designed experiments. One data set, produced by GNF (San Diego, CA) contained 24 time points representing samples collected every 2 hours, while the other two (produced by PBRC, Baton Rouge LA and Harvard Medical School) contained 12 time points taken every 4 hours.

**Figure 2:**
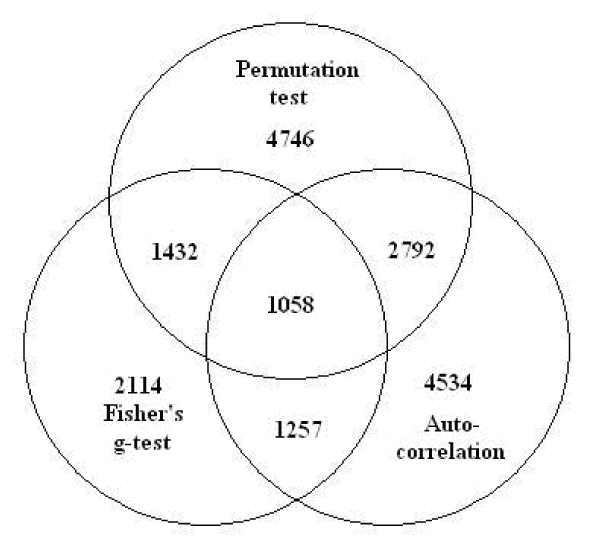
**The number of oscillating genes revealed by three different algorithms**. 5400 genes are reported by at least one algorithm. The most number of oscillating genes is revealed by Pt-test.

Analysis of the locally produced murine liver data revealed more circadially oscillating genes than it anticipated from previous publications [4,5]. We applied 3 different algorithms; the total number of circadian oscillating genes revealed by at least one of the algorithms was 5400 out of total 22690 genes (~23%). The largest number of oscillating genes (4746) is reported by Pt-test, followed by autocorrelation and Fisher's g-test, similar to the results on the simulated data. The Venn diagram on Figure 2 shows general agreement between the results of all the algorithms, particularly between the autocorrelation and Pt-test.

**Figure 3:**
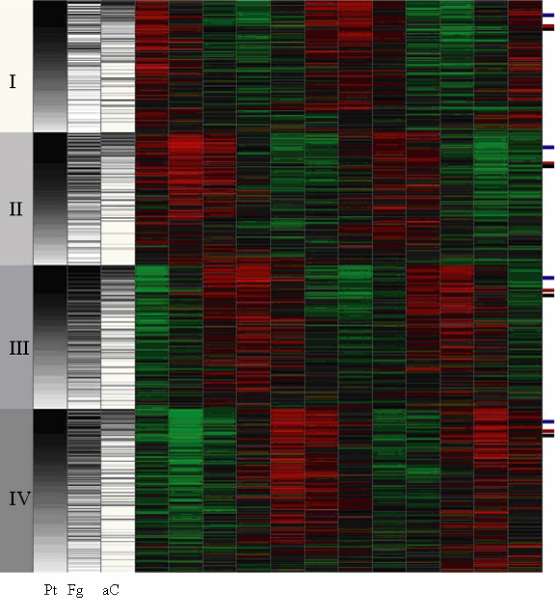
**Summary of the microarray analysis of circadian periodicity and phase in murine liver**. Summary of the microarray analysis of circadian periodicity and phase in murine liver. The Roman numerals represent the grouping of all expressed genes based on the calculated circadian phase, displayed in circadian time. The gray shades of first 3 columns display the p-value ranging from 0 (black) to white (1). After phase assignment in each of four phase classes the non-randomness of periodic pattern is assessed by 3 different algorithms: Permutation test developed at PBRC (Pt), Autocorrelation (aC) and Fisher's g-test (Fg). When sorted by permutation test (Pt) p-value other algorithms generally agree with the permutation test selection, but offer less sensitivity. Out of over 22,690 genes presented on this heat map over 5400 (23.8%) are expressed with circadian periodicity above p < 0.05 cutoff as estimated by at least one of three algorithms. Tickmarks on the right side mark the 0.05 cutoff for 3 algorithms (Pt-test-black, Fg-test -blue, aC-red).

Another illustration of the performance and agreement between algorithms is presented on Figure 3. Here all 22690 gene expression profiles are sorted into 4 distinct groups by the phase of oscillation. To determine the phase we generate a series of ideal cosine curves with the same number of time points



(here *p *is the number of time points per one complete circadian period), each one with a phase shift by one time point (4 hours if related to PBRC data). We calculate a pair-wise correlation between gene expression profile and each phase of ideal cosine curve. The phase shift (lag) that produces the highest correlation coefficient is assumed to be the most likely phase of a particular profile. This method is superior to assigning the phase by position of the zenith and nadir, as it takes into account ascending and descending trends in the whole data sets rather than single points. Within each phase, expression profiles are sorted by ascending order of p-value estimated in Pt-test, depicted as gray scale in the first column. The next 2 columns, Fg and aC represent p-values estimated through Fisher's g-test and autocorrelation analysis, respectively. Although they do not follow the same exact order, both these columns display similar patterns as the Pt column. The heatmap shows a characteristic pattern of two red zones (elevated gene expression) spaced by dark or green areas (reduced gene expression) prominent in all phases. In each phase group (i.e. expression profiles with highest correlation to a cosine curve with the same phase shift) the pattern shows some deterioration close to the bottom, but never disappears completely. This test suggests that the permutation is likely to be a correct, although still conservative, estimation of the true number of circadially expressed genes in murine liver.

**Figure 4:**
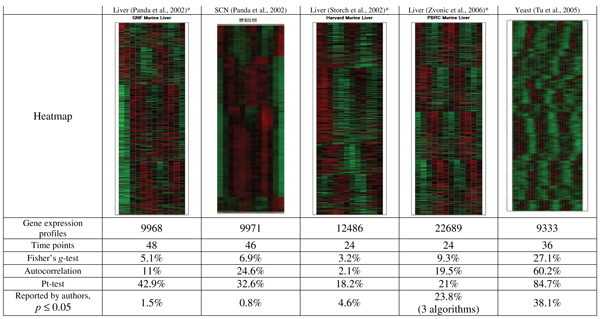
**Analysis of circadian periodicity in tree murine liver data sets by thee algorithms**. Analysis of circadian periodicity in tree murine liver data sets by thee algorithms. The heatmaps present all genes in each data set grouped by phase shift and sorted by Pt-test p-value, similar to Figure 2. The last row shows the fraction of oscillating genes as reported in the original publications and supplementary materials. Data from columns marked * has been previously published in [4]

In both external data sets (Storch et al. and Panda et al., referred here as Harvard and GNF data set respectively) we have apparently identified more periodically expressed genes than was previously reported by the authors. The results of the analysis are summarized in Figure 4. In data sets of 12 time points [5] and [3]) all three algorithms revealed that 20–25% of all genes followed a circadian rhythm with confidence levels of at least 95%. It should be also noted that previously reported 5 to 15% of periodically-expressed genes was calculated in relation to the estimated number of active genes, which in turn is influenced by arbitrary selection criteria. We report both the absolute numbers and percentage of periodically expressed genes in relation to all genes present in the data set. Analysis of a larger data set of 24 time points [6] produced radically different results. Fisher's g-test revealed 511 oscillating genes with p < 0.05, or ~5% of all genes examined (Figure 4). The autocorrelation method identified 1278 or ~13% of all genes oscillating with circadian periodicity. In contrast to the first two methods, our permutation test reported a circadian pattern in ~80% of all genes. Although these values were larger than previously reported in liver [7,8,3], they may still underestimate the actual number of oscillating genes. The circadian pattern (i.e. two red zones of elevated gene expression spaced by two dark or green zones of decreased expression over two circadian periods) is prominent in all thee heatmaps included in Figure 4. This pattern extends beyond the selected p < 0.05 cutoff.

## Discussion

It is likely that most genes following the circadian pattern are not linked directly to the basic circadian clock mechanism. Rather, they are modulated by the downstream circadian effectors, some of which can be responsive to the supply of energy and macronutrients, which, in turn, is closely linked to the daily feeding pattern. Connections between basic circadian mechanisms and nutrient homeostasis have been demonstrated [9]. Specifically, at least two components of the basic circadian pacemaker, *Bmal1 *and *Clock*, were found to regulate glucose levels and thus, play a significant role in the energy balance. This finding is consistent with the accepted view that circadian clocks are important in driving activity and feeding behavior in mammals. This may also explain the difference between heatmaps presented on Figure 4. The phase groups on the heatmap of PBRC data set are approximately equal and two peaks are evenly spaced. The other two data sets have more expression profiles with poorly defined or unevenly spaced peaks and phase groups are obviously different in size. At the PBRC samples were collected from mice maintained under constant diurnal 12 hr light: 12 hr dark environment, while the two other data sets were produced under conditions simulating constant darkness. Storch et al. have entrained the mice to a 12 h light/darkness cycle and then collected the samples after the animals were transferred to a constant dim light environment. Panda et al. entrained the mice to a 12 h light/darkness cycle and then collected the samples after several days of complete darkness. These differences in experimental design, specifically with respect to photic stimuli, may explain the expression patterns we describe. This includes phase shifts and deregulated amplitude in the expression patterns for many genes, although not a complete loss of oscillation. Our Pt-test analysis of the largest collection of time series expression profiles in peripheral tissues [6] leads to a conclusion that the majority of all expressed genes are affected by the circadian rhythm. Direct application of the established methods for identification of circadian expressed genes to our data already revealed a larger portion of oscillating genes relative to previous reports, 20–25% vs. 5–15%. It should be also taken into account that we relate the number of revealed oscillating genes to the total unfiltered number of genes in the data set, no matter how low their expression level may be. If the same method had been applied to the external data sets, the oscillating fraction of genes reported by the original authors would have been reduced to <5%.

In addition to circadian data we have analyzed a different time series microarray data, recently published by Tu et al., [10]. This data represents gene expression of *Sacharomices cerevisae *measured at 36 consecutive time points. The original authors report an oscillating pattern with a period of ~300 *min *in a large part of expressed genes. This ultradian oscillating pattern reflects temporal compartmentalization of components of yeast metabolic cycle. Our analysis of the same data corroborates the prominence of oscillation reported in the original paper. However, our approach allows identification of oscillating pattern in even larger portion of expression profiles. Consistently with all other experiments in summarized in Figure 4, the Pt-test identifies the largest number of oscillating genes, 7911 instead of 3552 reported by Tu et al. This number includes practically all actively expressed genes, which is logical and expectable since the authors have postulated that the whole energy supply of the yeast cell oscillates, imposing the rhythm on all cellular processes.

## Conclusion

From the analysis of both simulated and real data from independent sources we conclude that Pt-test consistently reveals more circadially oscillating genes in short time series compared to the other tested algorithms and generally less sensitive to stochastic non-periodic noise. The drawback of this algorithm is that it is much more computationally demanding compared to autocorrelation or Fisher's g-test. However, its effective C++ implementation allows processing of realistic amounts of data in a reasonable time on an average personal computer. Typically, analysis of a complete data set takes less then an hour, which is trivial compared to the time it takes to collect the data. The source code of Pt-test procedure, as well as computationally effective C++ implementations of autocorrelation, DFT and Fisher's g-test procedures are free and available from the authors upon request.

## Methods

### Time series data sets

#### Pennington data set

We have completed independent circadian studies in AKR/J mice acclimated to a 12 hr light: 12 hr dark cycle, harvesting sets of 3–5 mice at 4 hr intervals in duplicates over a 24 hr period [5]. Total RNA samples from inguinal (iWAT) white adipose tissue, brown adipose tissue (BAT), and liver have been assayed by Affymetrix microarrays. A few genes have been selected for validation with RT-PCR for the expression profile of representative circadian rhythm genes in all 3 tissues. The transcriptomic data set contained over 22,000 gene expression profiles for each of 3 different tissues. In the current study, we have used only the murine liver data. Since each time point was sampled twice, the following Fourier transform for each profile can be re-arranged into a short time series that represents two complete circadian cycles. Profiles have been smoothened by a 3^rd ^degree polynomial procedure and median-subtracted. For better compatibility, the same smoothing and median subtraction procedure has been applied to all other data sets.

#### GNF data set

This data set was provided courtesy of Dr. Hogenesch [6] and contains microarray expression profiles of nearly 10,000 genes in murine liver and hypothalamus, measured at 2 hr intervals over a 48 hr period. Our analysis used only the liver subset.

#### Harvard data set

This data set, provided courtesy of Dr. Storch [3], was collected from murine heart and liver; only the liver data set was used in our study. Each data set has 12 time points collected at even intervals of 4 hours over a period of 48 hours. The experimental design is similar to our own based on number of time points and the period of observation.

#### Yeast data set

This data has been kindly provided by Dr. Tu and Dr. McKnight [10]. The data contains microarray expression time series of 36 samples collected at intervals of ~25 *min*. It has been reported that the whole time series covers approximately three periods of 300 *min *each and the majority of expressed genes follow this oscillating pattern.

#### Simulated data set

To test and benchmark our algorithm we have generated an artificial data set based on a widely accepted model of periodic gene expression [11]:

*Y*_*t *_= *β *cos(*ωt *+ *φ*) + *ε*_*t*_,

where *β *is a positive constant, *ω *∈ *(*0, *π)*, *φ *uniformly distributed in (-*π*, *π*] and where *ε*_*t *_is a sequence of uncorrelated random variables with mean 0 and variance *σ*^2^, independent of *φ*. Since we use this data to test the ability of different algorithms to identify periodicity in gene expression profiles regardless of phase, we assume *φ *= 0 for all simulated profiles. In order to simulate different signal to noise ratios we also assume the amplitude for baseline variation constant, but add different noise component *ε *for individual profiles. We have generated 10000 sinusoidal expression profiles with amplitude 1000. The entire simulated data is subdivided into 10 categories (1000 profiles each). The *ε *value for each fraction was taken as a random number *ε*_*t *_∈ [0, 50* *i*], *i *= 10, 20, ...,100. To control for possible false positive oscillation we have reproduced each category with the same noise component *ε*, but with an absent baseline oscillation, i.e. amplitude *φ *= 0. The resulting data set is similar in number of profiles to the natural microarray data sets. Each profile consists of 12 data points, which is the same as the majority of published circadian microarray data sets, including two out of the three data sets used in this study.

### Algorithms

#### Data pre-processing

Profiles have been smoothened by a 3^rd ^degree polynomial procedure and median-subtracted. For smoothing we use seven-point Savitzky-Golay algorithm [12]. To take advantage of all points in the time series a single-pass smoothing has been applied in a circular manner, with the last points contributing to smoothing the starting points. For better compatibility, the same smoothing and median subtraction procedure has been applied to all data sets.

#### Spectral Analysis

For purposes of spectral analysis, consider a series of microarray expression values for gene *x *with *N *samples of the form

*Y *= *x*_0_, *x*_1_, *x*_2_, ...*x*_*N*-1_

This series can be converted from time-domain, where each variable represents a measurement in time to a frequency domain using Discrete Fourier Transform (DFT) algorithm. Frequency domain representation of the series of experiments is also known as periodogram, which can be denoted by *I*(*ω*):



If a time series has a significant sinusoidal component with frequency *ω *∈ [0, *π*], then the periodogram exhibits a peak at that frequency with a high probability. Conversely, if the time series is a purely random process (a.k.a "white noise"), then the plot of the periodogram against the Fourier frequencies approaches a straight line [13].

#### Fisher's g-test

The significance of the observed periodicity can be estimated by Fisher *g*-statistics, as recently recommended in [11]. Fisher derived an exact test of the maximum periodogram coordinate by introducing the *g*-statistic



where *I*(*ω*_*k*_) is a *k*-th peak of the periodogram. Large values of g indicate a non-random periodicity. We calculate the *p*-value of the test under the null hypothesis with the exact distribution of *g *using the following formula:



where *n *= [*N*/2] and *p *is the largest integer less than 1/*x*.

This algorithm closely follows the guidelines recommended for analysis of periodicities in time-series microarray data [11] with the exception that we applied a locally developed C++ code instead of R scripts.

#### Autocorrelation

For a discrete time series *Y *= *x*_0_, *x*_1_, *x*_2_, ...*x*_*N*-1 _the autocorrelation is simply the correlation of the expression profile against itself with a frame shift of *k *data points (where 0 ≤ *k *≤ *N *- 1, often referred as the lag). For the time shift *f*, defined as *f *= *i *+ *k *if *i*+*k*<*N *and *f *= *i *+ *k *- *N *otherwise



For each time series we calculate the maximum positive *R(f) *among all possible phase shifts *f *and use tabulates 0.05 significance cutoff values for correlation coefficient. Time series that shows significant autocorrelation *R(f) *with the lag *f *corresponding to one day (6 data points) are considered circadially expressed.

## Authors' contributions

SZ and JMG has conceived the experiments and produced the data for analysis. AAP has developed the algorithms, the software and performed data analysis and algorithm testing and benchmarking. AAP and JMG wrote the paper.
